# Trends in foodborne outbreaks and outbreak-associated illnesses using a Bayesian trend model – United States, 1998–2018

**DOI:** 10.1371/journal.pone.0353943

**Published:** 2026-08-04

**Authors:** Michael C. Bazaco, Michael Batz, Andrea Cote, LaTonia Richardson, Iva Bilanovic, Robert M. Hoekstra, Joanna Zablotsky Kufel, Beau Bruce

**Affiliations:** 1 US Food and Drug Administration Human Foods Program, College Park, Maryland, United States of America; 2 US Department of Agriculture Food Safety Inspection Service, Washington, District of Columbia, United States of America; 3 US Centers for Disease Control and Prevention, Atlanta, Georgia, United States of America; University of Illinois Urbana-Champaign College of Veterinary Medicine, UNITED STATES OF AMERICA

## Abstract

Evaluating temporal trends in foodborne outbreak and illness attribution to specific food types is important for understanding which foods may be emerging as sources of illness and whether prevention strategies are working to prevent these illnesses. Evaluating these trends can be difficult because foodborne outbreaks are uncommon, and attribution of these outbreaks to a specific food can be difficult. We present a Bayesian trend model looking at outbreaks and outbreak-associated illnesses linked to four Interagency Food Safety Analytics Collaboration (IFSAC) priority foodborne bacterial pathogens: *Campylobacter*, Shiga toxin-producing *Escherichia coli* O157, *Listeria monocytogenes*, and *Salmonella* using a categorization scheme IFSAC created to classify foods into 17 categories that closely align with the U.S. food regulatory agencies’ classification needs. Outbreaks and outbreak-associated illnesses of listeriosis increased in dairy products during 1998–2018 while those linked to meat and poultry decreased. Outbreaks of campylobacteriosis linked to both dairy and poultry products also increased. STEC O157 outbreaks and outbreak-associated illnesses linked to meat decreased over the period. Outbreaks and outbreak-associated illnesses of salmonellosis linked to eggs also decreased over the period. This new approach to evaluating temporal changes in outbreaks and outbreak-associated illnesses may be a useful tool in understanding the epidemiology and impacts of prevention strategies for foodborne illness.

## Introduction

Evaluating temporal trends in foodborne outbreaks and illnesses attributed to specific food types is important for understanding emerging sources of foodborne illness and the impact of interventions. In recent years, the U.S. Food and Drug Administration (FDA) and U.S. Department of Agriculture Food Safety and Inspection Service (FSIS) have implemented new guidance, standards, and rules to prevent foodborne illness and strengthen food safety. Some, such as the FDA’s Prevention of *Salmonella* Enteritidis in Shell Eggs During Production, Transportation, and Storage (74 FR 33030) or “Egg Rule”; the declaration by FSIS of Shiga toxin-producing *Escherichia coli* O157 (STEC O157) adulterants in raw beef products (9 CFR CH3, docket no. 97-068N), and FSIS’ performance standards for chicken have resulted in substantial reductions on foodborne illness [1–3]. Other rules, such as the implementation of various new rules under the Food Safety Modernization Act (FSMA) have the potential to further reduce this burden [4,5].

The Interagency Food Safety Analytics Collaboration (IFSAC) works to improve U.S. foodborne illness source attribution estimates, with a focus on *Salmonella*, *Campylobacter*, *Listeria monocytogenes (L. monocytogenes)*, and STEC O157, through a collaborative working group including the U.S. Centers for Disease Control and Prevention (CDC), FDA, and FSIS. IFSAC considers these four pathogens to be priorities because of the frequency and severity of illness they cause, and because targeted interventions can significantly reduce these illnesses. As such, this analysis only focused on these 4 pathogens.

IFSAC utilizes a hierarchical categorization scheme, which consists of 17 categories and a five-level hierarchy for categorizing implicated foods [6]. Level 1 reflects the broadest categories and includes aquatic animals, land animals, and plants. Level 2 further classifies the Level 1 categories into more specific animals (dairy, game, meat-poultry, egg) or botanical (grains-beans, nuts-seeds, oils-sugars, produce) food categories. Levels 3–5 classify the foods into increasingly specific categories, with the most specific categories reflecting the food processing, preparation, or consumption type (e.g., ready-to-eat meats and canned/containerized produce). Each of the subcategories also has an unknown category for outbreaks which cannot be assigned at that level of specificity. For example, outbreaks known to be from a plant-based food, but for which the specificity is inadequate to assign to only one of the grains-beans, nuts-seeds, oils-sugars, or produce subcategories (such as a sauce that includes onions, beans, olive oil, and peppers) would be assigned to Plants at Level 1, but to ‘Unknown’ at more specific levels of the hierarchy.

We applied a Bayesian negative binomial regression model to evaluate temporal trends of the number of outbreaks and outbreak-associated illnesses for four pathogens, attributed to specific food categories at three levels of hierarchy. We also developed an accompanying R package, ifsac.trendr (https://github.com/cdcgov/ifsac.trendr), to facilitate the estimates and visualize these results. The model allows us to evaluate both the magnitude and significance of changes in foodborne illness burden across multiple levels of granularity of food categorization for these pathogens. Applying the model across our hierarchical categorization also allows us to assess what specific food products may be driving changes in more general categories.

This analysis is distinct from that presented in IFSAC’s annual reports of foodborne illness source attribution estimates. Those reports provide attribution estimates based on statistical modeling of multiple years of outbreak data; the time periods used in these estimates are overlapping as each update incorporates another year of outbreak data. As a result of this and other methodological factors, annual report point estimates should not be directly compared across years. To address this challenge, IFSAC developed this Bayesian model to provide a methodologically sound approach to evaluate trends in outbreaks and outbreak associated illnesses over time, which are inputs used in the development of annual attribution estimates.

## Methods

### Data

State, local, and territorial health departments report foodborne disease outbreaks to CDC through the Foodborne Disease Outbreak Surveillance System (FDOSS). All data used in this analysis was extracted from this system. It should be noted that this system is dynamic, so data extraction at different times may result in different results. CDC defines a foodborne disease outbreak as the occurrence of 2 or more cases of a similar illness resulting from the ingestion of a common food. We extracted outbreak data from FDOSS on reported foodborne outbreaks during 1998–2018 caused or suspected to be caused by nontyphoidal *Salmonella*, STEC O157, *L. monocytogenes*, and *Campylobacter*. We included outbreaks with a single causal pathogen (multi-pathogen outbreaks were excluded), and for which implicated foods could be assigned to one of 17 IFSAC categories. Outbreaks involving multiple foods were excluded. Outbreaks involving multi-ingredient foods were only included if a single contaminated ingredient was confirmed as the source of the outbreak or when all the ingredients in a multi-ingredient food could be classified into one of our categories (fruit salad consisting only of various fruits).

We analyzed trends in the number of foodborne illness outbreaks and confirmed outbreak-associated illnesses attributed to the 17 categories that compose the first two hierarchical levels (Levels 1 and 2, hereafter the “top-level” categories) for each of the four pathogens [6].

### Model development

We sought to achieve several goals in the development of our trend model. In our previous attribution models, a log transformation of outbreak size was performed to mitigate the impact of large outbreaks and to enable the incorporation of additional variables before being back-transformed to calculate attribution estimate percentages [6]. Since this analysis focuses on the raw counts of outbreaks and outbreak associated illnesses and not calculating attribution percentages, we used a negative binomial outcome distribution with a log link function to address overdispersion common to surveillance count data. To mitigate against the influence of intermittent large outbreaks, we used thin plate splines to apply a data-driven smoothing of the data over time. To report various levels of statistical uncertainty over time and to have the ability to easily evaluate multiple comparisons between different periods, we chose a Bayesian approach.

### Statistical analysis

We implemented our Bayesian negative binomial regression model using thin plate splines with the R package bamlss (v. 1.0–0) using defaults unless otherwise noted in R (v. 3.4.2) [7]. We fit models on two outcomes: outbreak count and number of outbreak-associated illnesses. [6]. The right-hand side of our model consisted only of our thin plate spline smoothing function on year. Individual models were run for each of the four pathogens at Level 1 of the IFSAC categorization scheme (4 pathogens x 7 categories = 28 models) and Level 2 of the IFSAC categorization scheme (4 pathogens x 16 categories [includes Unknown subcategories] = 64 models) for each of the two outcomes resulting in a total of 184 models. Models were fit using 20,000 Markov Chain Monte Carlo (MCMC) iterations with 5,000 burn-in iterations and thinned every 100 steps. Predictions of the mean value of the trend were computed using the samples from the fitting process. Summary quantiles (0.025, 0.05, 0.25, 0.5, 0.75, 0.95, 0.975) were calculated for each year on the resulting distribution of trends. Pairwise differences in the predicted mean value of the outcome were calculated for each combination of 1-, 2-, 3-, 5/6-, and 10/11-year windows and were considered significant if zero was excluded at the 0.025 and 0.975 quantiles of the distribution of these differences, allowing for comparison of any time windows, regardless of adjacency. Fitting problems were detected automatically (e.g., failure to converge or means outside the confidence bounds) and were excluded from further evaluation. The full results of the analysis at each level can be found in Appendix 1. Here we present results of the 5- and 6-year time windows (1998–2003, 2003–2008, 2009–2013, 2014–2018).

**Fig 1:**
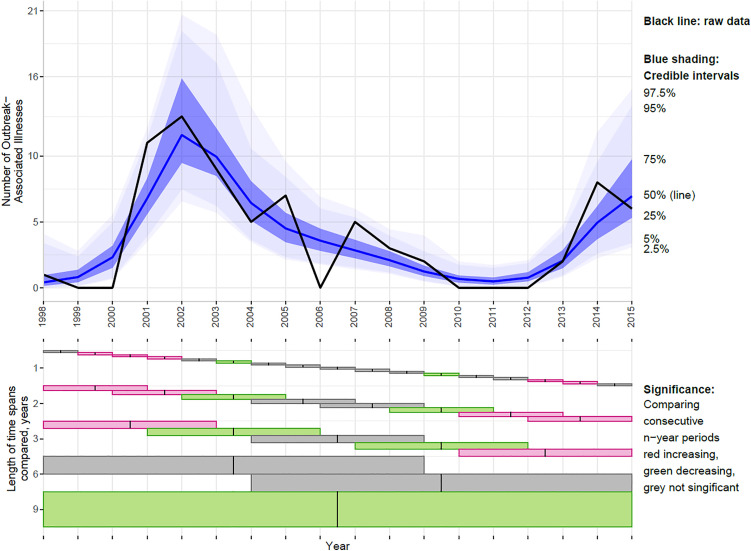
Overview of trend model visualization.

A custom visualization was developed to display the fitted model with its associated uncertainty and the significance of pairwise comparisons between various consecutive periods (Fig 1). For additional pairwise comparisons, tables were automatically constructed which highlighted significant differences using the same color scheme as the visualization. An R package, ifsac.trendr, is available for the modeling and its associated visualization and tabulation (https://github.com/cdcgov/ifsac.trendr).

## Results

We analyzed four pathogens across all IFSAC food categories at three levels of granularity. While there are too many analyses and visualizations to include in the manuscript, the full set can be found within an interactive visualization tool here (Appendix 1 Link), which allows the user to drill down through different levels of the food hierarchy and view point estimates and credibility intervals for each comparison at all time window comparison levels. Below we present five examples that show significant temporal changes in outbreak or outbreak-associated illnesses at one or more levels of the food hierarchy and over different timeframes. For some of the pathogen food pairs not included here, no significant changes are identified using this model in the evaluated time period.

### *L. monocytogenes* in land animal foods

**Fig 2:**
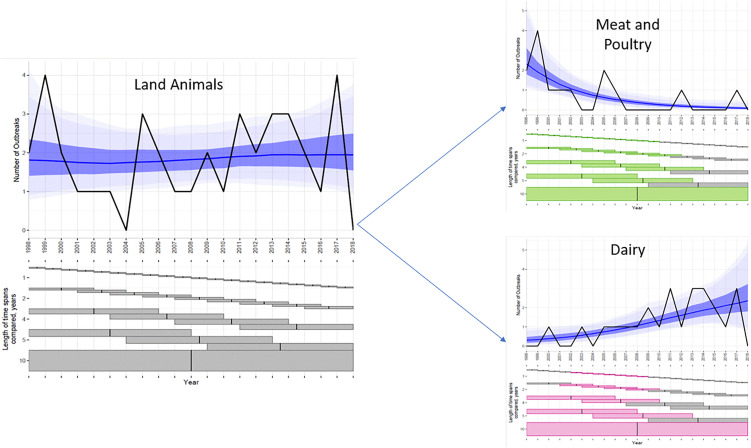
L. monocytogenes outbreaks in products from land animals.

The number of outbreaks of listeriosis linked to foods of land animal origin did not show any significant change over time (Fig 2). However, changes were seen in more specific subcategories. When looking at dairy outbreaks of listeriosis, a significant increase is evident, particularly in the middle portion of the evaluation period. There was a median of 0.4 (0.1–1.1) more annual outbreaks linked to dairy when comparing the second (2004–2008) time window to the first (1998–2003), a 1.0 (0.3–2.2) annual outbreak increase when comparing the third (2009–2013) to the first, and a 1.7 (0.4–3.7) annual outbreak increase between the fourth (2014–2018) and the first. On the other hand, listeriosis outbreaks linked to meat and poultry significantly decreased from 1998 through 2018, with a decrease of 1.0 (0.3–2.2) outbreaks when comparing the second time window to the first, a decrease of 1.3 (0.5–2.6) between the third and first, and a decrease of 1.4 (0.6–2.7) between the fourth and first.

**Fig 3:**
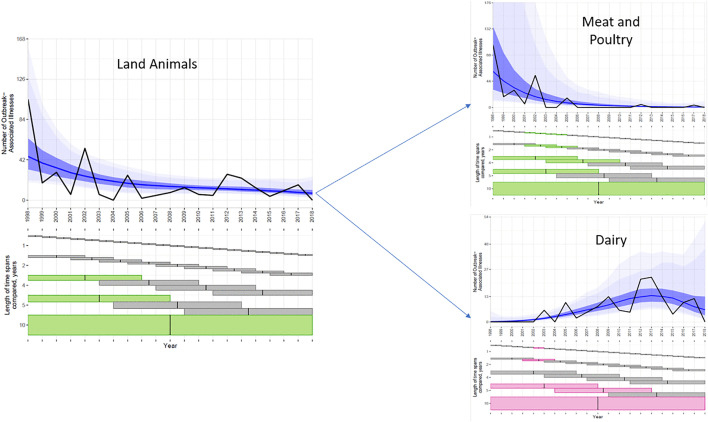
*L. monocytogenes* outbreak-associated Illnesses from land animals.

There was a significant decrease in outbreak related illnesses of listeriosis associated with foods of animal origin in the early part of the period evaluated (Fig 3). Particularly, there was a median significant decrease of 15.4 (1.1–56.6) illnesses annually between the first and second time period, a decrease of 20.5 (0.3–63.3) illnesses between the first and third, and a decrease of 23.4 (3.2–67.0) illnesses between the first and most recent period. This decrease did flatten out, however, and there was no significant decrease in the most recent time periods. Like results of the outbreak count analysis, the change was not seen consistently across all subcategories. Significant reductions were seen in the Meat-Poultry category (including foods such as beef, chicken, and turkey) with a median decrease of 26.0 (1.4–1215.6) annual illnesses between the earliest window and second window, a decrease of 30.6 (4.2–1270.1) illnesses between the first and third, and a decrease of 32.2 (5.4–1275.3) annual illnesses between the first and fourth window. Alternatively, there was a significant increase in illnesses associated with dairy products, with a median annual increase of 4.2 (0.4–10.4) annual illnesses between the first and the second window, 10.8 (4.5–28.5) between the first and third, and 9.6 (3.2–345.0) between the first and fourth. As with outbreaks, this increase was mostly driven by outbreak illnesses linked to solid/semi-solid dairy products, such as cheese and ice cream.

### *Campylobacter* in land animal foods

**Fig 4:**
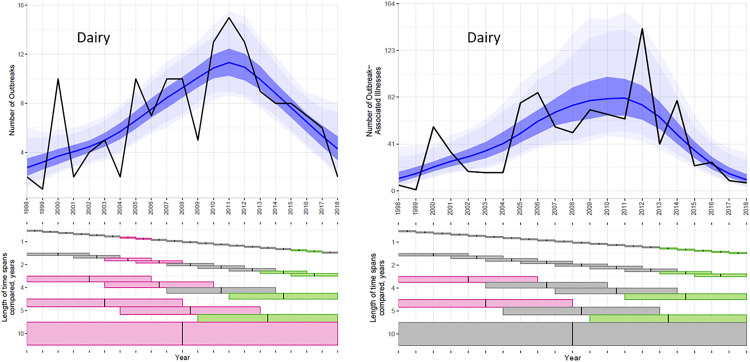
*Campylobacter* outbreaks and outbreak-associated illnesses from dairy.

**Fig 5:**
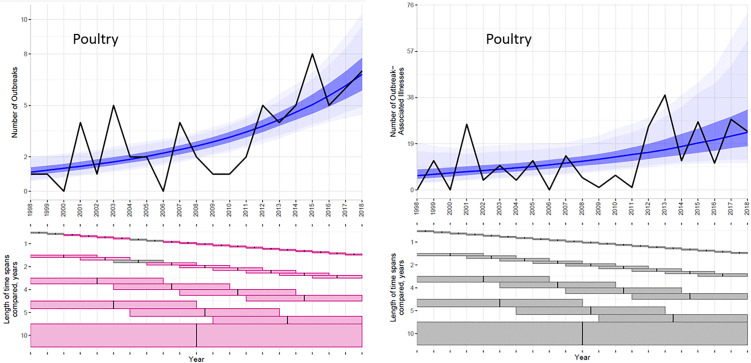
*Campylobacter* outbreaks and outbreak-associated illnesses from poultry.

*Campylobacter* outbreaks and illnesses linked to foods of land animal origin steadily increased over the time period. Poultry and dairy were the two major drivers. There was an early increase in outbreaks linked to dairy products, with a median increase of 3.7 (1.4–62) annual outbreaks per year when comparing the first time window to the second, an increase of 6.7 (3.7–9.9) annual outbreaks between the first and third, and an increase of 2.6 (0.2–5.5) annual outbreaks between the first and fourth. The outbreak numbers trended down later in the period with a significant median decrease of 4.2 (0.9–7.6) annual outbreaks per year when comparing the third and fourth time window (Fig 4). These changes were driven almost entirely by changes in both pasteurized and unpasteurized fluid milk. Outbreaks linked to poultry increased consistently over the time period. All time window comparisons showed a significant increase, most notably when comparing the first and most recent time period where a median annual increase of 4.2 (2.2–6.6) outbreaks was observed. The most recent window also showed a median increase of 2.2 (0.9–4.0) annual outbreaks when compared to the third window (Fig 5).

Campylobacteriosis illnesses associated with outbreaks linked to dairy products showed similar trends to outbreaks with an early increase followed by a later decrease (Fig 4). A median increase of 35.6 (10.9–73.1) annual outbreak illnesses per year was seen when comparing the first time window to the second, and an increase of 52.5 annual outbreak illnesses (17.4–109.7) between the first and third. The outbreak illness numbers trended down later in the window with a significant median decrease of 48.7 (15.7–102.7) annual outbreak illnesses per year when comparing the third and fourth window and a median decrease of 31.3 (0.5–72.4) annual outbreak illnesses per year when comparing second and fourth window. There were no significant changes in poultry outbreak illnesses over time, despite the changes noted in outbreak numbers (Fig 5).

### STEC O157 in beef

**Fig 6:**
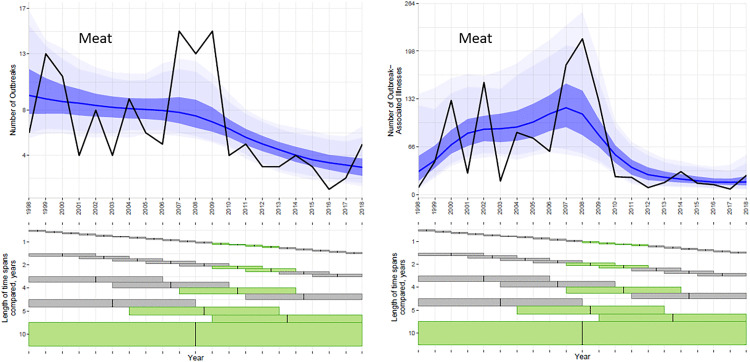
Shiga toxin-producing *Escherichia coli* O157 outbreaks and outbreak-associated illnesses from meat.

Outbreaks of STEC O157 linked to meat, the majority of which was beef, significantly decreased over time (Fig 6). This was most notable in the middle time window. When comparing the last window to the first, there was a median of 5.3 (1.9–10.0) fewer outbreaks annually, and 4.5 (1.5–9.0) fewer outbreaks when compared to the second window. This trend did hold through the final window as there was a median of 2.1 (0.4–4.5) fewer outbreaks in the final window compared to the third window.

Illnesses linked to these outbreaks similarly decreased over time (Fig 6). The most recent time window had a median of 51.0 (17.8–119.4) fewer illnesses than the first, 91.5 (34.2–170.2) fewer illnesses than the second, and 26 (3.0–61.4) fewer illnesses than the third window. There was also a significant median decrease of 63 (14.6–141) fewer illnesses between the second and third windows.

### *Salmonella* in eggs

**Fig 7:**
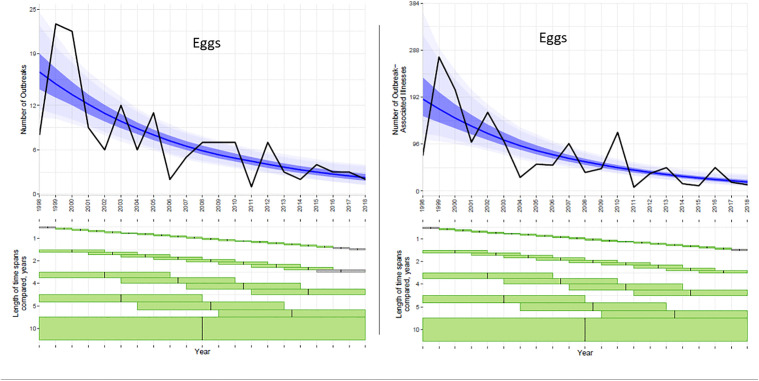
*Salmonella* outbreaks and outbreak-associated illnesses from eggs.

Outbreaks of salmonellosis linked to eggs decreased significantly and consistently throughout the overall 21-year period evaluated (Fig 7). All six of the comparisons between time windows showed significant decreases in median outbreaks. The most recent window had a median of 10.1 (5.8–15.5) fewer outbreaks than the first, 4.5 (2.4–6.7) fewer than the second and 1.7 (0.7–2.7) fewer than the third window.

The findings for outbreak numbers are consistent with what is seen in illnesses linked to those outbreaks (Fig 7). The most recent time window had a median of 120.9 (65.3–22.6) fewer illnesses than the first, 50.7 (28.0–77.7) fewer illnesses than the second, and 18.5 (9.6–30.2) fewer illnesses then the third. Significant decreases were seen in all six of the comparisons.

### *Salmonella* in meat and poultry

While the number of outbreaks of salmonellosis linked to meat and poultry did not increase over the overall 21-year period, the number of outbreak-associated illnesses did, particularly in more recent time windows. A significant increase in median annual outbreak illnesses of 318.3 (69.3–664.5) was noted when comparing the most recent time period to the first and an increase of 153.3 (3.6–419.0) was noted when compared to the third window. In poultry, a significant increase in foodborne outbreak illnesses of 175.0 (30.8–441.6) was seen comparing the most recent time window to the first, and an increase of 139.3 (15.9–382.2) illnesses compared to the second. For meat, the most recent time window had a significant increase of 139.7 outbreak illnesses (35.1–348.2) compared to the first window and an increase of 77.0 illnesses (3.3–263.9) compared to the third window.

## Discussion

Understanding changes over time in which foods are most often linked with foodborne illnesses enables us to identify emerging problems and evaluate interventions. Here we present an approach to estimate temporal changes in specific food categories linked to foodborne outbreak and outbreak-associated illnesses for four major pathogens between 1998 and 2018. This approach allows us to account for the overdispersion of the outbreak and illness data. We also show that, although no change may be evident at broader levels of categorization, significant changes existed at more granular subcategories, as seen in the results for *L. monocytogenes* and land animals. Our approach also provides multiple credibility intervals around these estimates, enabling better decision making for different circumstances and permits us to evaluate changes between different time periods. As noted, unlike the IFSAC annual attribution reports, the modeling approach presented here enables statistical comparisons of outbreaks and outbreak associated illnesses over time. As such, this analysis provides a complementary perspective for evaluating potential changes in foodborne illness attribution.

The findings of this analysis indicate that the frequency of outbreaks and outbreak-associated illnesses linked to food changed for some pathogen-food category pairs over time, such as the five examples presented in this article. Listeriosis outbreaks and outbreak-related illnesses linked to solid and semi-solid dairy products increased over the study period. This is consistent and continues a trend noted in a 2013 study by CDC [8]. This increase appears to be mostly driven by increases in the occurrence of outbreaks linked to solid/semi solid dairy products and rather than milk. Recent listeriosis outbreaks in 2021 and 2022 linked to cheese and ice cream indicate that this trend may be continuing [9–11]. Conversely, outbreaks and outbreak-related illnesses linked to ready-to-eat meat products decreased over the time period, consistent with previous findings [12,13]. Since 2019, outbreaks of listeriosis linked to deli meats have occurred, though most have been linked to various products sliced at deli counters where a particular deli meat was not identified [14–16]. FSIS even observed a substantial decrease in ready-to-eat (RTE) product positives from 0.7% in 2005 to about 0.2% in 2017 [17]. In 2010, FSIS conducted a risk assessment for *L. monocytogenes* in deli meats, and the industry implementation of effective *L. monocytogenes* mitigation interventions on the basis of the risk assessment have coincided with reductions in listeriosis associated with deli meats [18]. The source of outbreaks associated with delis that slice both meat and cheese can be difficult to identify due to cross-contamination of countertops, deli slicers, surfaces, hands, and the ability of *L. monocytogenes* to grow and survive at cold temperatures in a refrigerator [16]. More recently, from May to November 2024, a large outbreak of *L. monocytogenes* illnesses occurred that was linked to liverwurst product and possibly other ready-to-eat (RTE) products produced in the same facility. FSIS conducted a comprehensive review following the outbreak, which resulted in immediate sampling and inspection enhancements [19].

Foodborne outbreaks and outbreak-associated illnesses of campylobacteriosis increased during the last 20 years, particularly among foods of land animal origin. This was driven by increases in outbreaks linked to poultry and dairy products. Dairy product outbreaks increased early in the time frame, but leveled off in later years, and were almost entirely driven by outbreaks linked to fluid milk. Many of these outbreaks were associated with unpasteurized fluid milk, which is not commonly consumed by the general population [20,21]. Outbreaks of campylobacteriosis linked to poultry increased more consistently over the study period, but overall outbreak-associated illnesses remained steady throughout the time period. A disproportionate number of these outbreaks were associated with chicken liver products which, similar to raw milk, are not widely consumed by the general population. Chicken liver products are often consumed undercooked in the form of mousse and pâté [20,22,23].

Similarly, a significant decrease in both foodborne outbreaks and outbreak-associated *Salmonella* illnesses linked to eggs were noted over the time period. Williams *et al.* found similar trends when they looked at salmonellosis rates in the U.S., and these findings are also consistent with, and continue the pattern, noted by Minor and Parrett, who looked at outbreaks and outbreak-associated illnesses from 1998–2008 [1,24]. In 2004, the FDA submitted a proposed rule intended to reduce the burden of *Salmonella* illnesses coming from eggs entitled “Prevention of *Salmonella* Enteritidis in Shell Eggs During Production.” In the time frame from when the rule was proposed (2004) to the compliance dates (2010–2012), the egg industry likely began to develop and implement procedures to reduce *Salmonella* contamination of eggs, which may explain the reduction in outbreaks and outbreak-associated illnesses noted here. However, the rule does not apply to all egg producers.

There was a notable increase in foodborne outbreak-associated illnesses of *Salmonella* linked to meat and poultry in the most recent time period (2014–2018), though the total number of outbreaks did not increase. While this may indicate the occurrence of larger outbreaks, whole genome sequencing (WGS) implementation also occurred in that time period; making this trend a potential artifact of improved resolution in foodborne illness investigations.

Outbreaks of STEC O157 linked to the meat category decreased over the time period, particularly in the middle of the time frame. The same trends were noted in outbreak-associated illnesses. This category is mostly represented by beef. In 1994, FSIS declared STEC O157 an adulterant in raw ground beef products and instituted a sampling program to reduce the pathogen in ground beef and create a safer product for consumers. Reductions in the rate of illnesses from STEC O157 in meat products indicate that declaring STEC O157 an adulterant in raw ground beef—and industry efforts to respond to this rule—have been effective [25,26].

Food safety agencies recommend meat and poultry products be prepared to appropriate temperatures, and interventions, specifically those targeting specific foods such as raw eggs and beef, may have contributed to a lower number of illnesses attributed to these foods [27]. Despite the guidance, there are still food items, such as chicken livers, that often are consumed raw or undercooked. Further, foods that are often consumed raw, such as produce and juices, have increasingly been reported as sources of foodborne illnesses [28–31]. However, quantifying changes over time in the priority ranking of those sources can be challenging due to the small numbers of outbreaks and the fact that sparsity is not uniform across all pathogen food pairs and categories. Further, quantifying changes in outbreak size is complicated by recent technological advancements in pathogen identification, including the replacement of pulsed-field gel electrophoresis (PFGE) by the higher resolution WGS in both regulatory sampling and outbreak investigations [32,33]. For example, the formalization of Sample-Initiated Retrospective Outbreak investigations may be increasing both the size and number of outbreaks [34]. Recent analysis of other enteric illness outbreak data indicates there has been a marked increase in overall outbreak counts between 2009–2019, though much of this may have been a result of the inclusion of person-to-person outbreaks [35].

When evaluating the impacts of targeted interventions or new regulatory actions, examining various time windows with significance testing is helpful as there may be variable lag periods before changes can be identified.

Due to the nature of foodborne outbreaks, the goal of this analysis is to provide trend estimates at a level of granularity useful to developing food safety strategies. Each model is independent of each other and, therefore, multiplicity of testing and statistical power issues may still exist. However, we do provide credibility intervals for comparison of time periods. Additionally, our findings line up, temporally, with regulatory changes which support these findings. For example, the model was generally not able to fit data where there is less than one outbreak per year on average. Also, even at the level of specificity we were able to achieve, we still lack the ability to model specific foods (e.g., tomatoes vs. cucumbers in the seeded vegetable category) that may have different risk profiles and contribute differently to their broader category’s changes. However, many model categories with few outbreaks per year are also less likely to be of interest since they are not reported to be causing many outbreaks. In addition, it is important to note that many outbreaks are never attributed to a single food source and, therefore, were not included in this analysis. This is a global limitation of analyzing foodborne illness outbreaks.

This model builds on previous work evaluating such trends by mitigating the influence of large outbreaks using new smoothing functions [36]. It also uses untransformed data, which makes the findings easier to explain, communicate, and compare with other data sources. This work can serve as a starting point for further research into the development of improved models that could incorporate other epidemiologic factors that may influence illness counts, such as single or multi-state designations, venues, or points of contamination. Alternative outcome distributions and approaches (e.g., penalization) can be explored to improve the model, especially the upper bounds of confidence limits for rarer counts like those seen for *L. monocytogenes.*

The model utilized in this analysis further allows us to test changes in attribution between specific time points or time windows. This versatile tool can also be applied to other over-dispersed count data, both within and outside of food safety, such as evaluating outbreak events related to waterborne pathogens or the seasonality of vector-borne illnesses or non-infectious hazards, such as environmental exposures.

A common characteristic of the analyses comparing trends in both outbreaks and outbreak-associated illnesses (Fig 4-7) is that significant trends are more frequently observed when modeling outbreaks. This result demonstrates that statistical tests based on outbreak counts are inherently more powerful than analyses based on outbreak-associated illnesses [37].

## Conclusion

The findings presented here may help inform regulatory agencies on significant trends in foodborne outbreak data, evaluate interventions or policies, and detect emerging food safety issues. Lastly, the model developed here can be robustly applied to other data where overdispersion and paucity of data are a limitation in the future.

## Supporting information

S1 File S9 Full Output of all model comparisons across all levels of food categorization. (ZIP)
