# Supplementary material for: Trends in foodborne outbreaks and outbreak-associated illnesses using a Bayesian trend model – United States, 1998–2018
Source: PLoS One. 2026 Aug 4;21(8):e0353943. doi: 10.1371/journal.pone.0353943 (PMC13436697; doi:10.1371/journal.pone.0353943)
Supplement: Supplementary file 1 [file pone.0353943.s001.zip › S9 Full Model Output across all food comparisons.html]

Project 23 Analysis


# Project 23 Analysis

#### Beau B. Bruce, lue7 at cdc dot gov, EDEB/DFWED/NCEZID/CDC

#### January 07, 2022

# Introduction

This report details the results of IFSAC project 23's use of a Bayesian model to evaluate change over time in IFSAC's four priority pathogens *Campylobacter*, *E coli* O157, *Listeria monocytogenes*, and *Salmonella* within the IFSAC project 6 food categorization scheme at the top three levels. So far, we have evaluated changes in the number of outbreaks (`OutbreakCount`) and confirmed primary illnesses (`ConfirmedPrimary`) from 1998 to 2015.

The document is organized hierarchically (top to bottom) by outcome measure, IFSAC categorization level, pathogen-food category. Use the table of contents on the left to navigate between sections.

Each major section includes a summary plot. For each pathogen-food category pair, there is a plot and a pairwise comparison of all 1-, 2-, 4-, 5-, and 10-year groups (organized in tab group) with significant changes in highlighted in green (decreasing) or red (increasing). On the plots, the black line represents the observed data, the blue line the median prediction of the model with three shades of blue representing (darkest to lightest) the interquartile range (25th to 75th percetile, 50% credible interval), the 5th/95th percentile (90% credible interval), and the 2.5th/97.5th percentile (95% credible interval) of the mean prediction for the outcome variable.

# Outbreak Count

## IFSAC Level 1

### Campylobacter:Aquatic Animals

#### Year group pairwise comparisons

##### 1-year

##### 2-year

##### 4-year

##### 5-year

##### 10-year

### Campylobacter:Land Animals

#### Year group pairwise comparisons

##### 1-year

##### 2-year

##### 4-year

##### 5-year

##### 10-year

### Campylobacter:Multiple Foods Reported

#### Year group pairwise comparisons

##### 1-year

##### 2-year

##### 4-year

##### 5-year

##### 10-year

### Campylobacter:Multiple-Ingredient Food

#### Year group pairwise comparisons

##### 1-year

##### 2-year

##### 4-year

##### 5-year

##### 10-year

### Campylobacter:Other

#### Year group pairwise comparisons

##### 1-year

##### 2-year

##### 4-year

##### 5-year

##### 10-year

### Campylobacter:Plant

#### Year group pairwise comparisons

##### 1-year

##### 2-year

##### 4-year

##### 5-year

##### 10-year

### Campylobacter:Unclassifiable

#### Year group pairwise comparisons

##### 1-year

##### 2-year

##### 4-year

##### 5-year

##### 10-year

### Escherichia:Aquatic Animals

#### Year group pairwise comparisons

##### 1-year

##### 2-year

##### 4-year

##### 5-year

##### 10-year

### Escherichia:Land Animals

#### Year group pairwise comparisons

##### 1-year

##### 2-year

##### 4-year

##### 5-year

##### 10-year

### Escherichia:Multiple Foods Reported

#### Year group pairwise comparisons

##### 1-year

##### 2-year

##### 4-year

##### 5-year

##### 10-year

### Escherichia:Multiple-Ingredient Food

#### Year group pairwise comparisons

##### 1-year

##### 2-year

##### 4-year

##### 5-year

##### 10-year

### Escherichia:Other

#### Year group pairwise comparisons

##### 1-year

##### 2-year

##### 4-year

##### 5-year

##### 10-year

### Escherichia:Plant

#### Year group pairwise comparisons

##### 1-year

##### 2-year

##### 4-year

##### 5-year

##### 10-year

### Escherichia:Unclassifiable

#### Year group pairwise comparisons

##### 1-year

##### 2-year

##### 4-year

##### 5-year

##### 10-year

### Listeria:Aquatic Animals

#### Year group pairwise comparisons

##### 1-year

##### 2-year

##### 4-year

##### 5-year

##### 10-year

### Listeria:Land Animals

#### Year group pairwise comparisons

##### 1-year

##### 2-year

##### 4-year

##### 5-year

##### 10-year

### Listeria:Multiple-Ingredient Food

#### Year group pairwise comparisons

##### 1-year

##### 2-year

##### 4-year

##### 5-year

##### 10-year

### Listeria:Plant

#### Year group pairwise comparisons

##### 1-year

##### 2-year

##### 4-year

##### 5-year

##### 10-year

### Salmonella:Aquatic Animals

#### Year group pairwise comparisons

##### 1-year

##### 2-year

##### 4-year

##### 5-year

##### 10-year

### Salmonella:Land Animals

#### Year group pairwise comparisons

##### 1-year

##### 2-year

##### 4-year

##### 5-year

##### 10-year

### Salmonella:Multiple Foods Reported

#### Year group pairwise comparisons

##### 1-year

##### 2-year

##### 4-year

##### 5-year

##### 10-year

### Salmonella:Multiple-Ingredient Food

#### Year group pairwise comparisons

##### 1-year

##### 2-year

##### 4-year

##### 5-year

##### 10-year

### Salmonella:Other

#### Year group pairwise comparisons

##### 1-year

##### 2-year

##### 4-year

##### 5-year

##### 10-year

### Salmonella:Plant

#### Year group pairwise comparisons

##### 1-year

##### 2-year

##### 4-year

##### 5-year

##### 10-year

### Salmonella:Seeded vegetables

#### Year group pairwise comparisons

##### 1-year

##### 2-year

##### 4-year

##### 5-year

##### 10-year

### Salmonella:Unclassifiable

#### Year group pairwise comparisons

##### 1-year

##### 2-year

##### 4-year

##### 5-year

##### 10-year

## IFSAC Level 2

### Campylobacter:Aquatic Animals:Fish

#### Year group pairwise comparisons

##### 1-year

##### 2-year

##### 4-year

##### 5-year

##### 10-year

### Campylobacter:Aquatic Animals:Shell Fish

#### Year group pairwise comparisons

##### 1-year

##### 2-year

##### 4-year

##### 5-year

##### 10-year

### Campylobacter:Land Animals:Dairy

#### Year group pairwise comparisons

##### 1-year

##### 2-year

##### 4-year

##### 5-year

##### 10-year

### Campylobacter:Land Animals:Game

#### Year group pairwise comparisons

##### 1-year

##### 2-year

##### 4-year

##### 5-year

##### 10-year

### Campylobacter:Land Animals:Meat-Poultry

#### Year group pairwise comparisons

##### 1-year

##### 2-year

##### 4-year

##### 5-year

##### 10-year

### Campylobacter:Land Animals:Unknown

#### Year group pairwise comparisons

##### 1-year

##### 2-year

##### 4-year

##### 5-year

##### 10-year

### Campylobacter:Multiple Foods Reported:Unknown

#### Year group pairwise comparisons

##### 1-year

##### 2-year

##### 4-year

##### 5-year

##### 10-year

### Campylobacter:Multiple-Ingredient Food:Unknown

#### Year group pairwise comparisons

##### 1-year

##### 2-year

##### 4-year

##### 5-year

##### 10-year

### Campylobacter:Other:Unknown

#### Year group pairwise comparisons

##### 1-year

##### 2-year

##### 4-year

##### 5-year

##### 10-year

### Campylobacter:Plant:Oils-Sugars

#### Year group pairwise comparisons

##### 1-year

##### 2-year

##### 4-year

##### 5-year

##### 10-year

### Campylobacter:Plant:Produce

#### Year group pairwise comparisons

##### 1-year

##### 2-year

##### 4-year

##### 5-year

##### 10-year

### Campylobacter:Unclassifiable:Unknown

#### Year group pairwise comparisons

##### 1-year

##### 2-year

##### 4-year

##### 5-year

##### 10-year

### Escherichia:Aquatic Animals:Fish

#### Year group pairwise comparisons

##### 1-year

##### 2-year

##### 4-year

##### 5-year

##### 10-year

### Escherichia:Aquatic Animals:Unknown

#### Year group pairwise comparisons

##### 1-year

##### 2-year

##### 4-year

##### 5-year

##### 10-year

### Escherichia:Land Animals:Dairy

#### Year group pairwise comparisons

##### 1-year

##### 2-year

##### 4-year

##### 5-year

##### 10-year

### Escherichia:Land Animals:Game

#### Year group pairwise comparisons

##### 1-year

##### 2-year

##### 4-year

##### 5-year

##### 10-year

### Escherichia:Land Animals:Meat-Poultry

#### Year group pairwise comparisons

##### 1-year

##### 2-year

##### 4-year

##### 5-year

##### 10-year

### Escherichia:Land Animals:Multiple Land Animals

#### Year group pairwise comparisons

##### 1-year

##### 2-year

##### 4-year

##### 5-year

##### 10-year

### Escherichia:Multiple Foods Reported:Unknown

#### Year group pairwise comparisons

##### 1-year

##### 2-year

##### 4-year

##### 5-year

##### 10-year

### Escherichia:Multiple-Ingredient Food:Unknown

#### Year group pairwise comparisons

##### 1-year

##### 2-year

##### 4-year

##### 5-year

##### 10-year

### Escherichia:Other:Unknown

#### Year group pairwise comparisons

##### 1-year

##### 2-year

##### 4-year

##### 5-year

##### 10-year

### Escherichia:Plant:Grains-Beans

#### Year group pairwise comparisons

##### 1-year

##### 2-year

##### 4-year

##### 5-year

##### 10-year

### Escherichia:Plant:Nuts-Seeds

#### Year group pairwise comparisons

##### 1-year

##### 2-year

##### 4-year

##### 5-year

##### 10-year

### Escherichia:Plant:Other Plant

#### Year group pairwise comparisons

##### 1-year

##### 2-year

##### 4-year

##### 5-year

##### 10-year

### Escherichia:Plant:Produce

#### Year group pairwise comparisons

##### 1-year

##### 2-year

##### 4-year

##### 5-year

##### 10-year

### Escherichia:Unclassifiable:Unknown

#### Year group pairwise comparisons

##### 1-year

##### 2-year

##### 4-year

##### 5-year

##### 10-year

### Listeria:Aquatic Animals:Fish

#### Year group pairwise comparisons

##### 1-year

##### 2-year

##### 4-year

##### 5-year

##### 10-year

### Listeria:Land Animals:Dairy

#### Year group pairwise comparisons

##### 1-year

##### 2-year

##### 4-year

##### 5-year

##### 10-year

### Listeria:Land Animals:Meat-Poultry

#### Year group pairwise comparisons

##### 1-year

##### 2-year

##### 4-year

##### 5-year

##### 10-year

### Listeria:Multiple-Ingredient Food:Unknown

#### Year group pairwise comparisons

##### 1-year

##### 2-year

##### 4-year

##### 5-year

##### 10-year

### Listeria:Plant:Produce

#### Year group pairwise comparisons

##### 1-year

##### 2-year

##### 4-year

##### 5-year

##### 10-year

### Listeria:Plant:Unknown

#### Year group pairwise comparisons

##### 1-year

##### 2-year

##### 4-year

##### 5-year

##### 10-year

### Salmonella:Aquatic Animals:Fish

#### Year group pairwise comparisons

##### 1-year

##### 2-year

##### 4-year

##### 5-year

##### 10-year

### Salmonella:Aquatic Animals:Other Aquatic Animals

#### Year group pairwise comparisons

##### 1-year

##### 2-year

##### 4-year

##### 5-year

##### 10-year

### Salmonella:Aquatic Animals:Shell Fish

#### Year group pairwise comparisons

##### 1-year

##### 2-year

##### 4-year

##### 5-year

##### 10-year

### Salmonella:Land Animals:Dairy

#### Year group pairwise comparisons

##### 1-year

##### 2-year

##### 4-year

##### 5-year

##### 10-year

### Salmonella:Land Animals:Eggs

#### Year group pairwise comparisons

##### 1-year

##### 2-year

##### 4-year

##### 5-year

##### 10-year

### Salmonella:Land Animals:Game

#### Year group pairwise comparisons

##### 1-year

##### 2-year

##### 4-year

##### 5-year

##### 10-year

### Salmonella:Land Animals:Meat-Poultry

#### Year group pairwise comparisons

##### 1-year

##### 2-year

##### 4-year

##### 5-year

##### 10-year

### Salmonella:Land Animals:Multiple Land Animals

#### Year group pairwise comparisons

##### 1-year

##### 2-year

##### 4-year

##### 5-year

##### 10-year

### Salmonella:Land Animals:Unknown

#### Year group pairwise comparisons

##### 1-year

##### 2-year

##### 4-year

##### 5-year

##### 10-year

### Salmonella:Multiple Foods Reported:Unknown

#### Year group pairwise comparisons

##### 1-year

##### 2-year

##### 4-year

##### 5-year

##### 10-year

### Salmonella:Multiple-Ingredient Food:Unknown

#### Year group pairwise comparisons

##### 1-year

##### 2-year

##### 4-year

##### 5-year

##### 10-year

### Salmonella:Other:Unknown

#### Year group pairwise comparisons

##### 1-year

##### 2-year

##### 4-year

##### 5-year

##### 10-year

### Salmonella:Plant:Grains-Beans

#### Year group pairwise comparisons

##### 1-year

##### 2-year

##### 4-year

##### 5-year

##### 10-year

### Salmonella:Plant:Nuts-Seeds

#### Year group pairwise comparisons

##### 1-year

##### 2-year

##### 4-year

##### 5-year

##### 10-year

### Salmonella:Plant:Oils-Sugars

#### Year group pairwise comparisons

##### 1-year

##### 2-year

##### 4-year

##### 5-year

##### 10-year

### Salmonella:Plant:Other Plant

#### Year group pairwise comparisons

##### 1-year

##### 2-year

##### 4-year

##### 5-year

##### 10-year

### Salmonella:Plant:Produce

#### Year group pairwise comparisons

##### 1-year

##### 2-year

##### 4-year

##### 5-year

##### 10-year

### Salmonella:Plant:Unknown

#### Year group pairwise comparisons

##### 1-year

##### 2-year

##### 4-year

##### 5-year

##### 10-year

### Salmonella:Seeded vegetables:Seeded vegetables

#### Year group pairwise comparisons

##### 1-year

##### 2-year

##### 4-year

##### 5-year

##### 10-year

### Salmonella:Unclassifiable:Unknown

#### Year group pairwise comparisons

##### 1-year

##### 2-year

##### 4-year

##### 5-year

##### 10-year

## IFSAC Level 3

### Campylobacter:Aquatic Animals:Fish:Unknown

#### Year group pairwise comparisons

##### 1-year

##### 2-year

##### 4-year

##### 5-year

##### 10-year

### Campylobacter:Aquatic Animals:Shell Fish:Mollusks

#### Year group pairwise comparisons

##### 1-year

##### 2-year

##### 4-year

##### 5-year

##### 10-year

### Campylobacter:Land Animals:Dairy:Fluid milk

#### Year group pairwise comparisons

##### 1-year

##### 2-year

##### 4-year

##### 5-year

##### 10-year

### Campylobacter:Land Animals:Dairy:Solid/semi-solid dairy products

#### Year group pairwise comparisons

##### 1-year

##### 2-year

##### 4-year

##### 5-year

##### 10-year

### Campylobacter:Land Animals:Dairy:Unknown

#### Year group pairwise comparisons

##### 1-year

##### 2-year

##### 4-year

##### 5-year

##### 10-year

### Campylobacter:Land Animals:Game:Unknown

#### Year group pairwise comparisons

##### 1-year

##### 2-year

##### 4-year

##### 5-year

##### 10-year

### Campylobacter:Land Animals:Meat-Poultry:Meat

#### Year group pairwise comparisons

##### 1-year

##### 2-year

##### 4-year

##### 5-year

##### 10-year

### Campylobacter:Land Animals:Meat-Poultry:Poultry

#### Year group pairwise comparisons

##### 1-year

##### 2-year

##### 4-year

##### 5-year

##### 10-year

### Campylobacter:Land Animals:Meat-Poultry:Unknown

#### Year group pairwise comparisons

##### 1-year

##### 2-year

##### 4-year

##### 5-year

##### 10-year

### Campylobacter:Land Animals:Unknown:Unknown

#### Year group pairwise comparisons

##### 1-year

##### 2-year

##### 4-year

##### 5-year

##### 10-year

### Campylobacter:Multiple Foods Reported:Unknown:Unknown

#### Year group pairwise comparisons

##### 1-year

##### 2-year

##### 4-year

##### 5-year

##### 10-year

### Campylobacter:Multiple-Ingredient Food:Unknown:Unknown

#### Year group pairwise comparisons

##### 1-year

##### 2-year

##### 4-year

##### 5-year

##### 10-year

### Campylobacter:Other:Unknown:Unknown

#### Year group pairwise comparisons

##### 1-year

##### 2-year

##### 4-year

##### 5-year

##### 10-year

### Campylobacter:Plant:Oils-Sugars:Unknown

#### Year group pairwise comparisons

##### 1-year

##### 2-year

##### 4-year

##### 5-year

##### 10-year

### Campylobacter:Plant:Produce:Fruits

#### Year group pairwise comparisons

##### 1-year

##### 2-year

##### 4-year

##### 5-year

##### 10-year

### Campylobacter:Plant:Produce:Unknown

#### Year group pairwise comparisons

##### 1-year

##### 2-year

##### 4-year

##### 5-year

##### 10-year

### Campylobacter:Plant:Produce:Vegetables

#### Year group pairwise comparisons

##### 1-year

##### 2-year

##### 4-year

##### 5-year

##### 10-year

### Campylobacter:Unclassifiable:Unknown:Unknown

#### Year group pairwise comparisons

##### 1-year

##### 2-year

##### 4-year

##### 5-year

##### 10-year

### Escherichia:Aquatic Animals:Fish:Unknown

#### Year group pairwise comparisons

##### 1-year

##### 2-year

##### 4-year

##### 5-year

##### 10-year

### Escherichia:Aquatic Animals:Unknown:Unknown

#### Year group pairwise comparisons

##### 1-year

##### 2-year

##### 4-year

##### 5-year

##### 10-year

### Escherichia:Land Animals:Dairy:Fluid milk

#### Year group pairwise comparisons

##### 1-year

##### 2-year

##### 4-year

##### 5-year

##### 10-year

### Escherichia:Land Animals:Dairy:Solid/semi-solid dairy products

#### Year group pairwise comparisons

##### 1-year

##### 2-year

##### 4-year

##### 5-year

##### 10-year

### Escherichia:Land Animals:Dairy:Unknown

#### Year group pairwise comparisons

##### 1-year

##### 2-year

##### 4-year

##### 5-year

##### 10-year

### Escherichia:Land Animals:Game:Unknown

#### Year group pairwise comparisons

##### 1-year

##### 2-year

##### 4-year

##### 5-year

##### 10-year

### Escherichia:Land Animals:Meat-Poultry:Meat

#### Year group pairwise comparisons

##### 1-year

##### 2-year

##### 4-year

##### 5-year

##### 10-year

### Escherichia:Land Animals:Meat-Poultry:Poultry

#### Year group pairwise comparisons

##### 1-year

##### 2-year

##### 4-year

##### 5-year

##### 10-year

### Escherichia:Land Animals:Meat-Poultry:Unknown

#### Year group pairwise comparisons

##### 1-year

##### 2-year

##### 4-year

##### 5-year

##### 10-year

### Escherichia:Land Animals:Multiple Land Animals:Unknown

#### Year group pairwise comparisons

##### 1-year

##### 2-year

##### 4-year

##### 5-year

##### 10-year

### Escherichia:Multiple Foods Reported:Unknown:Unknown

#### Year group pairwise comparisons

##### 1-year

##### 2-year

##### 4-year

##### 5-year

##### 10-year

### Escherichia:Multiple-Ingredient Food:Unknown:Unknown

#### Year group pairwise comparisons

##### 1-year

##### 2-year

##### 4-year

##### 5-year

##### 10-year

### Escherichia:Other:Unknown:Unknown

#### Year group pairwise comparisons

##### 1-year

##### 2-year

##### 4-year

##### 5-year

##### 10-year

### Escherichia:Plant:Grains-Beans:Grains

#### Year group pairwise comparisons

##### 1-year

##### 2-year

##### 4-year

##### 5-year

##### 10-year

### Escherichia:Plant:Nuts-Seeds:Nuts

#### Year group pairwise comparisons

##### 1-year

##### 2-year

##### 4-year

##### 5-year

##### 10-year

### Escherichia:Plant:Other Plant:Unknown

#### Year group pairwise comparisons

##### 1-year

##### 2-year

##### 4-year

##### 5-year

##### 10-year

### Escherichia:Plant:Produce:Fruits

#### Year group pairwise comparisons

##### 1-year

##### 2-year

##### 4-year

##### 5-year

##### 10-year

### Escherichia:Plant:Produce:Unknown

#### Year group pairwise comparisons

##### 1-year

##### 2-year

##### 4-year

##### 5-year

##### 10-year

### Escherichia:Plant:Produce:Vegetables

#### Year group pairwise comparisons

##### 1-year

##### 2-year

##### 4-year

##### 5-year

##### 10-year

### Escherichia:Unclassifiable:Unknown:Unknown

#### Year group pairwise comparisons

##### 1-year

##### 2-year

##### 4-year

##### 5-year

##### 10-year

### Listeria:Aquatic Animals:Fish:Unknown

#### Year group pairwise comparisons

##### 1-year

##### 2-year

##### 4-year

##### 5-year

##### 10-year

### Listeria:Land Animals:Dairy:Fluid milk

#### Year group pairwise comparisons

##### 1-year

##### 2-year

##### 4-year

##### 5-year

##### 10-year

### Listeria:Land Animals:Dairy:Solid/semi-solid dairy products

#### Year group pairwise comparisons

##### 1-year

##### 2-year

##### 4-year

##### 5-year

##### 10-year

### Listeria:Land Animals:Meat-Poultry:Meat

#### Year group pairwise comparisons

##### 1-year

##### 2-year

##### 4-year

##### 5-year

##### 10-year

### Listeria:Land Animals:Meat-Poultry:Poultry

#### Year group pairwise comparisons

##### 1-year

##### 2-year

##### 4-year

##### 5-year

##### 10-year

### Listeria:Land Animals:Meat-Poultry:Unknown

#### Year group pairwise comparisons

##### 1-year

##### 2-year

##### 4-year

##### 5-year

##### 10-year

### Listeria:Multiple-Ingredient Food:Unknown:Unknown

#### Year group pairwise comparisons

##### 1-year

##### 2-year

##### 4-year

##### 5-year

##### 10-year

### Listeria:Plant:Produce:Fruits

#### Year group pairwise comparisons

##### 1-year

##### 2-year

##### 4-year

##### 5-year

##### 10-year

### Listeria:Plant:Produce:Vegetables

#### Year group pairwise comparisons

##### 1-year

##### 2-year

##### 4-year

##### 5-year

##### 10-year

### Listeria:Plant:Unknown:Unknown

#### Year group pairwise comparisons

##### 1-year

##### 2-year

##### 4-year

##### 5-year

##### 10-year

### Salmonella:Aquatic Animals:Fish:Other Fish

#### Year group pairwise comparisons

##### 1-year

##### 2-year

##### 4-year

##### 5-year

##### 10-year

### Salmonella:Aquatic Animals:Fish:Unknown

#### Year group pairwise comparisons

##### 1-year

##### 2-year

##### 4-year

##### 5-year

##### 10-year

### Salmonella:Aquatic Animals:Other Aquatic Animals:Unknown

#### Year group pairwise comparisons

##### 1-year

##### 2-year

##### 4-year

##### 5-year

##### 10-year

### Salmonella:Aquatic Animals:Shell Fish:Crustaceans

#### Year group pairwise comparisons

##### 1-year

##### 2-year

##### 4-year

##### 5-year

##### 10-year

### Salmonella:Aquatic Animals:Shell Fish:Mollusks

#### Year group pairwise comparisons

##### 1-year

##### 2-year

##### 4-year

##### 5-year

##### 10-year

### Salmonella:Aquatic Animals:Shell Fish:Unknown

#### Year group pairwise comparisons

##### 1-year

##### 2-year

##### 4-year

##### 5-year

##### 10-year

### Salmonella:Land Animals:Dairy:Fluid milk

#### Year group pairwise comparisons

##### 1-year

##### 2-year

##### 4-year

##### 5-year

##### 10-year

### Salmonella:Land Animals:Dairy:Powdered Dairy

#### Year group pairwise comparisons

##### 1-year

##### 2-year

##### 4-year

##### 5-year

##### 10-year

### Salmonella:Land Animals:Dairy:Solid/semi-solid dairy products

#### Year group pairwise comparisons

##### 1-year

##### 2-year

##### 4-year

##### 5-year

##### 10-year

### Salmonella:Land Animals:Dairy:Unknown

#### Year group pairwise comparisons

##### 1-year

##### 2-year

##### 4-year

##### 5-year

##### 10-year

### Salmonella:Land Animals:Eggs:Shell Eggs

#### Year group pairwise comparisons

##### 1-year

##### 2-year

##### 4-year

##### 5-year

##### 10-year

### Salmonella:Land Animals:Eggs:Unknown

#### Year group pairwise comparisons

##### 1-year

##### 2-year

##### 4-year

##### 5-year

##### 10-year

### Salmonella:Land Animals:Game:Unknown

#### Year group pairwise comparisons

##### 1-year

##### 2-year

##### 4-year

##### 5-year

##### 10-year

### Salmonella:Land Animals:Meat-Poultry:Meat

#### Year group pairwise comparisons

##### 1-year

##### 2-year

##### 4-year

##### 5-year

##### 10-year

### Salmonella:Land Animals:Meat-Poultry:Poultry

#### Year group pairwise comparisons

##### 1-year

##### 2-year

##### 4-year

##### 5-year

##### 10-year

### Salmonella:Land Animals:Meat-Poultry:Unknown

#### Year group pairwise comparisons

##### 1-year

##### 2-year

##### 4-year

##### 5-year

##### 10-year

### Salmonella:Land Animals:Multiple Land Animals:Unknown

#### Year group pairwise comparisons

##### 1-year

##### 2-year

##### 4-year

##### 5-year

##### 10-year

### Salmonella:Land Animals:Unknown:Unknown

#### Year group pairwise comparisons

##### 1-year

##### 2-year

##### 4-year

##### 5-year

##### 10-year

### Salmonella:Multiple Foods Reported:Unknown:Unknown

#### Year group pairwise comparisons

##### 1-year

##### 2-year

##### 4-year

##### 5-year

##### 10-year

### Salmonella:Multiple-Ingredient Food:Unknown:Unknown

#### Year group pairwise comparisons

##### 1-year

##### 2-year

##### 4-year

##### 5-year

##### 10-year

### Salmonella:Other:Unknown:Unknown

#### Year group pairwise comparisons

##### 1-year

##### 2-year

##### 4-year

##### 5-year

##### 10-year

### Salmonella:Plant:Grains-Beans:Beans

#### Year group pairwise comparisons

##### 1-year

##### 2-year

##### 4-year

##### 5-year

##### 10-year

### Salmonella:Plant:Grains-Beans:Grains

#### Year group pairwise comparisons

##### 1-year

##### 2-year

##### 4-year

##### 5-year

##### 10-year

### Salmonella:Plant:Grains-Beans:Unknown

#### Year group pairwise comparisons

##### 1-year

##### 2-year

##### 4-year

##### 5-year

##### 10-year

### Salmonella:Plant:Nuts-Seeds:Nuts

#### Year group pairwise comparisons

##### 1-year

##### 2-year

##### 4-year

##### 5-year

##### 10-year

### Salmonella:Plant:Nuts-Seeds:Seeds

#### Year group pairwise comparisons

##### 1-year

##### 2-year

##### 4-year

##### 5-year

##### 10-year

### Salmonella:Plant:Oils-Sugars:Unknown

#### Year group pairwise comparisons

##### 1-year

##### 2-year

##### 4-year

##### 5-year

##### 10-year

### Salmonella:Plant:Other Plant:Unknown

#### Year group pairwise comparisons

##### 1-year

##### 2-year

##### 4-year

##### 5-year

##### 10-year

### Salmonella:Plant:Produce:Fruits

#### Year group pairwise comparisons

##### 1-year

##### 2-year

##### 4-year

##### 5-year

##### 10-year

### Salmonella:Plant:Produce:Unknown

#### Year group pairwise comparisons

##### 1-year

##### 2-year

##### 4-year

##### 5-year

##### 10-year

### Salmonella:Plant:Produce:Vegetables

#### Year group pairwise comparisons

##### 1-year

##### 2-year

##### 4-year

##### 5-year

##### 10-year

### Salmonella:Plant:Unknown:Unknown

#### Year group pairwise comparisons

##### 1-year

##### 2-year

##### 4-year

##### 5-year

##### 10-year

### Salmonella:Seeded vegetables:Seeded vegetables:Seeded vegetables

#### Year group pairwise comparisons

##### 1-year

##### 2-year

##### 4-year

##### 5-year

##### 10-year

### Salmonella:Unclassifiable:Unknown:Unknown

#### Year group pairwise comparisons

##### 1-year

##### 2-year

##### 4-year

##### 5-year

##### 10-year

# Confirmed Primary Illnesses

## IFSAC Level 1

### Campylobacter:Aquatic Animals

#### Year group pairwise comparisons

##### 1-year

##### 2-year

##### 4-year

##### 5-year

##### 10-year

### Campylobacter:Land Animals

#### Year group pairwise comparisons

##### 1-year

##### 2-year

##### 4-year

##### 5-year

##### 10-year

### Campylobacter:Multiple Foods Reported

#### Year group pairwise comparisons

##### 1-year

##### 2-year

##### 4-year

##### 5-year

##### 10-year

### Campylobacter:Multiple-Ingredient Food

#### Year group pairwise comparisons

##### 1-year

##### 2-year

##### 4-year

##### 5-year

##### 10-year

### Campylobacter:Other

#### Year group pairwise comparisons

##### 1-year

##### 2-year

##### 4-year

##### 5-year

##### 10-year

### Campylobacter:Plant

#### Year group pairwise comparisons

##### 1-year

##### 2-year

##### 4-year

##### 5-year

##### 10-year

### Campylobacter:Unclassifiable

#### Year group pairwise comparisons

##### 1-year

##### 2-year

##### 4-year

##### 5-year

##### 10-year

### Escherichia:Aquatic Animals

#### Year group pairwise comparisons

##### 1-year

##### 2-year

##### 4-year

##### 5-year

##### 10-year

### Escherichia:Land Animals

#### Year group pairwise comparisons

##### 1-year

##### 2-year

##### 4-year

##### 5-year

##### 10-year

### Escherichia:Multiple Foods Reported

#### Year group pairwise comparisons

##### 1-year

##### 2-year

##### 4-year

##### 5-year

##### 10-year

### Escherichia:Multiple-Ingredient Food

#### Year group pairwise comparisons

##### 1-year

##### 2-year

##### 4-year

##### 5-year

##### 10-year

### Escherichia:Other

#### Year group pairwise comparisons

##### 1-year

##### 2-year

##### 4-year

##### 5-year

##### 10-year

### Escherichia:Plant

#### Year group pairwise comparisons

##### 1-year

##### 2-year

##### 4-year

##### 5-year

##### 10-year

### Escherichia:Unclassifiable

#### Year group pairwise comparisons

##### 1-year

##### 2-year

##### 4-year

##### 5-year

##### 10-year

### Listeria:Aquatic Animals

#### Year group pairwise comparisons

##### 1-year

##### 2-year

##### 4-year

##### 5-year

##### 10-year

### Listeria:Land Animals

#### Year group pairwise comparisons

##### 1-year

##### 2-year

##### 4-year

##### 5-year

##### 10-year

### Listeria:Multiple-Ingredient Food

#### Year group pairwise comparisons

##### 1-year

##### 2-year

##### 4-year

##### 5-year

##### 10-year

### Listeria:Plant

#### Year group pairwise comparisons

##### 1-year

##### 2-year

##### 4-year

##### 5-year

##### 10-year

### Salmonella:Aquatic Animals

#### Year group pairwise comparisons

##### 1-year

##### 2-year

##### 4-year

##### 5-year

##### 10-year

### Salmonella:Land Animals

#### Year group pairwise comparisons

##### 1-year

##### 2-year

##### 4-year

##### 5-year

##### 10-year

### Salmonella:Multiple Foods Reported

#### Year group pairwise comparisons

##### 1-year

##### 2-year

##### 4-year

##### 5-year

##### 10-year

### Salmonella:Multiple-Ingredient Food

#### Year group pairwise comparisons

##### 1-year

##### 2-year

##### 4-year

##### 5-year

##### 10-year

### Salmonella:Other

#### Year group pairwise comparisons

##### 1-year

##### 2-year

##### 4-year

##### 5-year

##### 10-year

### Salmonella:Plant

#### Year group pairwise comparisons

##### 1-year

##### 2-year

##### 4-year

##### 5-year

##### 10-year

### Salmonella:Seeded vegetables

#### Year group pairwise comparisons

##### 1-year

##### 2-year

##### 4-year

##### 5-year

##### 10-year

### Salmonella:Unclassifiable

#### Year group pairwise comparisons

##### 1-year

##### 2-year

##### 4-year

##### 5-year

##### 10-year

## IFSAC Level 2

### Campylobacter:Aquatic Animals:Fish

#### Year group pairwise comparisons

##### 1-year

##### 2-year

##### 4-year

##### 5-year

##### 10-year

### Campylobacter:Aquatic Animals:Shell Fish

#### Year group pairwise comparisons

##### 1-year

##### 2-year

##### 4-year

##### 5-year

##### 10-year

### Campylobacter:Land Animals:Dairy

#### Year group pairwise comparisons

##### 1-year

##### 2-year

##### 4-year

##### 5-year

##### 10-year

### Campylobacter:Land Animals:Game

#### Year group pairwise comparisons

##### 1-year

##### 2-year

##### 4-year

##### 5-year

##### 10-year

### Campylobacter:Land Animals:Meat-Poultry

#### Year group pairwise comparisons

##### 1-year

##### 2-year

##### 4-year

##### 5-year

##### 10-year

### Campylobacter:Land Animals:Unknown

#### Year group pairwise comparisons

##### 1-year

##### 2-year

##### 4-year

##### 5-year

##### 10-year

### Campylobacter:Multiple Foods Reported:Unknown

#### Year group pairwise comparisons

##### 1-year

##### 2-year

##### 4-year

##### 5-year

##### 10-year

### Campylobacter:Multiple-Ingredient Food:Unknown

#### Year group pairwise comparisons

##### 1-year

##### 2-year

##### 4-year

##### 5-year

##### 10-year

### Campylobacter:Other:Unknown

#### Year group pairwise comparisons

##### 1-year

##### 2-year

##### 4-year

##### 5-year

##### 10-year

### Campylobacter:Plant:Oils-Sugars

#### Year group pairwise comparisons

##### 1-year

##### 2-year

##### 4-year

##### 5-year

##### 10-year

### Campylobacter:Plant:Produce

#### Year group pairwise comparisons

##### 1-year

##### 2-year

##### 4-year

##### 5-year

##### 10-year

### Campylobacter:Unclassifiable:Unknown

#### Year group pairwise comparisons

##### 1-year

##### 2-year

##### 4-year

##### 5-year

##### 10-year

### Escherichia:Aquatic Animals:Fish

#### Year group pairwise comparisons

##### 1-year

##### 2-year

##### 4-year

##### 5-year

##### 10-year

### Escherichia:Aquatic Animals:Unknown

#### Year group pairwise comparisons

##### 1-year

##### 2-year

##### 4-year

##### 5-year

##### 10-year

### Escherichia:Land Animals:Dairy

#### Year group pairwise comparisons

##### 1-year

##### 2-year

##### 4-year

##### 5-year

##### 10-year

### Escherichia:Land Animals:Game

#### Year group pairwise comparisons

##### 1-year

##### 2-year

##### 4-year

##### 5-year

##### 10-year

### Escherichia:Land Animals:Meat-Poultry

#### Year group pairwise comparisons

##### 1-year

##### 2-year

##### 4-year

##### 5-year

##### 10-year

### Escherichia:Land Animals:Multiple Land Animals

#### Year group pairwise comparisons

##### 1-year

##### 2-year

##### 4-year

##### 5-year

##### 10-year

### Escherichia:Multiple Foods Reported:Unknown

#### Year group pairwise comparisons

##### 1-year

##### 2-year

##### 4-year

##### 5-year

##### 10-year

### Escherichia:Multiple-Ingredient Food:Unknown

#### Year group pairwise comparisons

##### 1-year

##### 2-year

##### 4-year

##### 5-year

##### 10-year

### Escherichia:Other:Unknown

#### Year group pairwise comparisons

##### 1-year

##### 2-year

##### 4-year

##### 5-year

##### 10-year

### Escherichia:Plant:Grains-Beans

#### Year group pairwise comparisons

##### 1-year

##### 2-year

##### 4-year

##### 5-year

##### 10-year

### Escherichia:Plant:Nuts-Seeds

#### Year group pairwise comparisons

##### 1-year

##### 2-year

##### 4-year

##### 5-year

##### 10-year

### Escherichia:Plant:Other Plant

#### Year group pairwise comparisons

##### 1-year

##### 2-year

##### 4-year

##### 5-year

##### 10-year

### Escherichia:Plant:Produce

#### Year group pairwise comparisons

##### 1-year

##### 2-year

##### 4-year

##### 5-year

##### 10-year

### Escherichia:Unclassifiable:Unknown

#### Year group pairwise comparisons

##### 1-year

##### 2-year

##### 4-year

##### 5-year

##### 10-year

### Listeria:Aquatic Animals:Fish
