# Supplementary material for: Trends in foodborne outbreaks and outbreak-associated illnesses using a Bayesian trend model – United States, 1998–2018
Source: PLoS One. 2026 Aug 4;21(8):e0353943. doi: 10.1371/journal.pone.0353943 (PMC13436697; doi:10.1371/journal.pone.0353943)
Supplement: Supplementary file 2 [file pone.0353943.s003.docx]

Dear Editorial Staff and Reviewers,

We are pleased to re-submit our manuscript entitled “Trends in foodborne outbreaks and outbreak-associated Illnesses using a Bayesian trend model – United States, 1998–2018” for continued consideration as a Research Article in PLOS ONE.

The comments and suggested changes to the manuscript have helped us craft a better manuscript with more clear and concise descriptions of our data sources, analysis inclusion and exclusion criteria, and limitations. We have also addressed issues with access to the code that is being used to model our data and produce the results presented.

Thank you so much for the time and effort you have put in reviewing our manuscript.

Please find specific responses to the review below (reviewer comments in italic):

-Michael C. Bazaco

*1.Please ensure that your manuscript meets PLOS ONE's style requirements, including those for file naming. The PLOS ONE style templates can be found at*

We have followed the style guide for the manuscript submission to the best of our understanding.

*2. When completing the data availability statement of the submission form, you indicated that you will make your data available on acceptance. We strongly recommend all authors decide on a data sharing plan before acceptance, as the process can be lengthy and hold up publication timelines. Please note that, though access restrictions are acceptable now, your entire data will need to be made freely accessible if your manuscript is accepted for publication. This policy applies to all data except where public deposition would breach compliance with the protocol approved by your research ethics board. If you are unable to adhere to our open data policy, please kindly revise your statement to explain your reasoning and we will seek the editor's input on an exemption. Please be assured that, once you have provided your new statement, the assessment of your exemption will not hold up the peer review process.*

Thank you for this clarification. When we chose this option, we were referring to the R code used for the model, so apologies for the confusion. All our data comes from the publicly available CDC Foodborne Disease Outbreak Surveillance System (FDOSS) and can be accessed at any time (<https://www.cdc.gov/nors/about/fdoss.html>). We have put a link to that data system into the manuscript (Line 76). In addition, we have made the R code for our modeling tool openly available as well. It is linked in the manuscript and that link is now live and open (Line 132).

*3. It appears that your figure captions and supporting figure captions are the same. Please confirm if the files were intended as figures or supporting figures. If so, kindly remove the supporting table captions in your manuscript.*

We were sure to upload the Figures as Figures, not Supporting Figures on resubmission and have removed the captions from the manuscript.

*4. We are unable to open your Supporting Information file “S8 Trends in FBO R Code Package.zip”. Please kindly revise as necessary and re-upload.*

We apologize for this. That attachment was the R package used to run the trend model described in the manuscript. In lieu of an attachment, we have made the R code public and embedded it into the revised manuscript draft (Line 132), attached.

*5. If the reviewer comments include a recommendation to cite specific previously published works, please review and evaluate these publications to determine whether they are relevant and should be cited. There is no requirement to cite these works unless the editor has indicated otherwise.*

This is not applicable for our resubmission.

*6. Please review your reference list to ensure that it is complete and correct. If you have cited papers that have been retracted, please include the rationale for doing so in the manuscript text, or remove these references and replace them with relevant current references. Any changes to the reference list should be mentioned in the rebuttal letter that accompanies your revised manuscript. If you need to cite a retracted article, indicate the article’s retracted status in the References list and also include a citation and full reference for the retraction notice.*

We will not be adding any additional references at resubmission.

*5. Review Comments to the Author*

*Reviewer #1: Only comment is quality of images before downloading is hard to read. For publication it should be ensured that the higher quality images are those to be included in the text. Otherwise research is sound.*

We are happy to work with the journal if there are any issues with the images during processing.

*Reviewer #2: GENERAL: This is an interesting and well written manuscript detailing time trend analysis of outbreaks and associated illnesses for four different pathogens. Good use of negative binomial regression and intelligent presentation of results. I had very few comments on the manuscript, most of which related to understanding what data weren't included in the analysis and overall interpretation.

SPECIFIC:
Line 90: This description about the difference between the modelling approach taken in this paper and the IFSAC annual reports is unclear. Suggest re-wording this to make it crystal clear how they are different and what the implications are.*

Thank you. We have added some additional language to better clarify the differences (lines 91-93)

*Line 103: Regarding the definition of foodborne outbreaks, were these two or more illnesses required to be microbiologically confirmed?*

These did not need to be laboratory confirmed. There did need to be confirmation of a common food source.

*Line 107: What happened to multipathogen outbreaks, which increased over time due to institution of culture independent diagnostic testing (CIDT) at a similar time to whole genome sequencing? It would also be good to mention the impact of CIDT in the discussion in relation to increased detection of associated cases.*

We note in the Data section of the methods that our analysis included only outbreaks where a single causal pathogen was identified. We have added some language to further emphasize that as well (Lines 80-81)

*Line 107: How did you handle outbreaks implicating multiple foods or poorly defined foods, such as a meal containing multiple ingredients.*

Outbreaks involving multiple foods were only included if a single contaminated ingredient was confirmed as the source of the outbreak or when all the ingredients in a multi-ingredient food could be classified into one of our categories (example fruit salad consisting only of various fruits). We have added some language to better explain that (Lines 82-85).

*Line 156: I would like to see a summary of the included datasets for each pathogen at the beginning of the results section. I feel like it is important to say what was excluded too so we understand the total dataset under consideration.*

All data used in this analysis came from the CDC Foodborne Disease Outbreak Surveillance System (FDOSS). We have added a note about this at the beginning of the Data section (Lines 76-78)

*Line 205: Is this comparison between third and fourth time period actually for each one against the first (reference) time period, which is how you would normal consider results in negative binomial regression. I note that these types of comparisons are made elsewhere, so good to clarify.*

This model was developed to allow for comparisons between any time period tested. This versatility is one of the strengths of the model, so comparisons are cited throughout, and indicated in the Figures.

*Line 264: You mention here trends in foodborne illnesses, but these are really 'foodborne disease outbreaks and associated illnesses', as most foodborne illnesses aren't linked to outbreaks.*

Thank you for this comment. We have adjusted the language surrounding the Minor and Parrett article, as well as the closing sentence of that paragraph (Lines 290-291 and Line 296)

*Line 328: You note the potential impact of whole genome sequencing for increased salmonella outbreaks, which is interesting. I would encourage further exploration of this issue (maybe not in this manuscript), as despite its usefulness data on effectiveness of whole genome sequencing is very hard to come by.*

Thank you for this comment. We agree that evaluating the continued impact of WGS (along with other tools such as CIDTs and metagenomics) in outbreak investigations and analysis is important and plan to continue to evaluate these impacts in future research.
