# Supplementary material for: Trends in foodborne outbreaks and outbreak-associated illnesses using a Bayesian trend model – United States, 1998–2018
Source: PLoS One. 2026 Aug 4;21(8):e0353943. doi: 10.1371/journal.pone.0353943 (PMC13436697; doi:10.1371/journal.pone.0353943)
Supplement: Peer review history

# Peer review: Decision Letter 0

**DOI:** 10.1371/journal.pone.0353943.r001

**Authors:** Csaba Varga

18 May 2026

PONE-D-26-18588

Trends in foodborne outbreaks and outbreak-associated illnesses using a Bayesian trend model – United States, 1998–2018

PLOS One

Dear Dr. Bazaco,

Thank you for submitting your manuscript to PLOS ONE. After careful consideration, we feel that it has merit but does not fully meet PLOS ONE’s publication criteria as it currently stands. Therefore, we invite you to submit a revised version of the manuscript that addresses the points raised during the review process.

Please submit your revised manuscript by Jul 02 2026 11:59PM. If you will need more time than this to complete your revisions, please reply to this message or contact the journal office at plosone@plos.org. When you're ready to submit your revision, log on to https://www.editorialmanager.com/pone/ and select the 'Submissions Needing Revision' folder to locate your manuscript file.

Please include the following items when submitting your revised manuscript:

- A letter that responds to each point raised by the academic editor and reviewer(s). You should upload this letter as a separate file labeled 'Response to Reviewers'.
- A marked-up copy of your manuscript that highlights changes made to the original version. You should upload this as a separate file labeled 'Revised Manuscript with Track Changes'.
- An unmarked version of your revised paper without tracked changes. You should upload this as a separate file labeled 'Manuscript'.

If you would like to make changes to your financial disclosure, please include your updated statement in your cover letter. Guidelines for resubmitting your figure files are available below the reviewer comments at the end of this letter.

If applicable, we recommend that you deposit your laboratory protocols in protocols.io to enhance the reproducibility of your results. Protocols.io assigns your protocol its own identifier (DOI) so that it can be cited independently in the future. For instructions see: https://journals.plos.org/plosone/s/submission-guidelines#loc-laboratory-protocols. Additionally, PLOS ONE offers an option for publishing peer-reviewed Lab Protocol articles, which describe protocols hosted on protocols.io. Read more information on sharing protocols at https://plos.org/protocols?utm_medium=editorial-email&utm_source=authorletters&utm_campaign=protocols.

As the corresponding author, your ORCID iD is verified in the submission system and will appear in the published article. PLOS supports the use of ORCID, and we encourage all coauthors to register for an ORCID iD and use it as well. Please encourage your coauthors to verify their ORCID iD within the submission system before final acceptance, as unverified ORCID iDs will not appear in the published article. *Only*  the individual author can complete the verification step; PLOS staff *cannot*  verify ORCID iDs on behalf of authors.

We look forward to receiving your revised manuscript.

Kind regards,

Csaba Varga, DVM MSc PhD

Academic Editor

PLOS One

Journal Requirements:

When submitting your revision, we need you to address these additional requirements.

1.Please ensure that your manuscript meets PLOS ONE's style requirements, including those for file naming. The PLOS ONE style templates can be found at

https://journals.plos.org/plosone/s/file?id=wjVg/PLOSOne_formatting_sample_main_body.pdf and

https://journals.plos.org/plosone/s/file?id=ba62/PLOSOne_formatting_sample_title_authors_affiliations.pdf

2. When completing the data availability statement of the submission form, you indicated that you will make your data available on acceptance. We strongly recommend all authors decide on a data sharing plan before acceptance, as the process can be lengthy and hold up publication timelines. Please note that, though access restrictions are acceptable now, your entire data will need to be made freely accessible if your manuscript is accepted for publication. This policy applies to all data except where public deposition would breach compliance with the protocol approved by your research ethics board. If you are unable to adhere to our open data policy, please kindly revise your statement to explain your reasoning and we will seek the editor's input on an exemption. Please be assured that, once you have provided your new statement, the assessment of your exemption will not hold up the peer review process.

3. It appears that your figure captions and supporting figure captions are the same. Please confirm if the files were intended as figures or supporting figures. If so, kindly remove the supporting table captions in your manuscript.

4. We are unable to open your Supporting Information file “S8 Trends in FBO R Code Package.zip”. Please kindly revise as necessary and re-upload.

5. If the reviewer comments include a recommendation to cite specific previously published works, please review and evaluate these publications to determine whether they are relevant and should be cited. There is no requirement to cite these works unless the editor has indicated otherwise.

6. Please review your reference list to ensure that it is complete and correct. If you have cited papers that have been retracted, please include the rationale for doing so in the manuscript text, or remove these references and replace them with relevant current references. Any changes to the reference list should be mentioned in the rebuttal letter that accompanies your revised manuscript. If you need to cite a retracted article, indicate the article’s retracted status in the References list and also include a citation and full reference for the retraction notice.

Reviewers' comments:

Reviewer's Responses to Questions

**Comments to the Author**

1. Is the manuscript technically sound, and do the data support the conclusions?

The manuscript must describe a technically sound piece of scientific research with data that supports the conclusions. Experiments must have been conducted rigorously, with appropriate controls, replication, and sample sizes. The conclusions must be drawn appropriately based on the data presented.

Reviewer #1: Yes

Reviewer #2: Yes

**********

2. Has the statistical analysis been performed appropriately and rigorously?

Reviewer #1: Yes

Reviewer #2: Yes

**********

3. Have the authors made all data underlying the findings in their manuscript fully available?

The PLOS Data policy requires authors to make all data underlying the findings described in their manuscript fully available without restriction, with rare exception (please refer to the Data Availability Statement in the manuscript PDF file). The data should be provided as part of the manuscript or its supporting information, or deposited to a public repository. For example, in addition to summary statistics, the data points behind means, medians and variance measures should be available. If there are restrictions on publicly sharing data—e.g. participant privacy or use of data from a third party—those must be specified.

Reviewer #1: Yes

Reviewer #2: Yes

**********

4. Is the manuscript presented in an intelligible fashion and written in standard English?

PLOS ONE does not copyedit accepted manuscripts, so the language in submitted articles must be clear, correct, and unambiguous. Any typographical or grammatical errors should be corrected at revision, so please note any specific errors here.

Reviewer #1: Yes

Reviewer #2: Yes

**********

5. Review Comments to the Author

Please use the space provided to explain your answers to the questions above. You may also include additional comments for the author, including concerns about dual publication, research ethics, or publication ethics. (Please upload your review as an attachment if it exceeds 20,000 characters)

Reviewer #1: Only comment is quality of images before downloading is hard to read. For publication it should be ensured that the higher quality images are those to be included in the text. Otherwise research is sound.

Reviewer #2: GENERAL: This is an interesting and well written manuscript detailing time trend analysis of outbreaks and associated illnesses for four different pathogens. Good use of negative binomial regression and intelligent presentation of results. I had very few comments on the manuscript, most of which related to understanding what data weren't included in the analysis and overall interpretation.

SPECIFIC:

Line 90: This description about the difference between the modelling approach taken in this paper and the IFSAC annual reports is unclear. Suggest re-wording this to make it crystal clear how they are different and what the implications are.

Line 103: Regarding the definition of foodborne outbreaks, were these two or more illnesses required to be microbiologically confirmed?

Line 107: What happened to multipathogen outbreaks, which increased over time due to institution of culture independent diagnostic testing (CIDT) at a similar time to whole genome sequencing? It would also be good to mention the impact of CIDT in the discussion in relation to increased detection of associated cases.

Line 107: How did you handle outbreaks implicating multiple foods or poorly defined foods, such as a meal containing multiple ingredients.

Line 156: I would like to see a summary of the included datasets for each pathogen at the beginning of the results section. I feel like it is important to say what was excluded too so we understand the total dataset under consideration.

Line 205: Is this comparison between third and fourth time period actually for each one against the first (reference) time period, which is how you would normal consider results in negative binomial regression. I note that these types of comparisons are made elsewhere, so good to clarify.

Line 264: You mention here trends in foodborne illnesses, but these are really 'foodborne disease outbreaks and associated illnesses', as most foodborne illnesses aren't linked to outbreaks.

Line 328: You note the potential impact of whole genome sequencing for increased salmonella outbreaks, which is interesting. I would encourage further exploration of this issue (maybe not in this manuscript), as despite its usefulness data on effectiveness of whole genome sequencing is very hard to come by.

**********

6. PLOS authors have the option to publish the peer review history of their article (what does this mean?). If published, this will include your full peer review and any attached files.

If you choose “no”, your identity will remain anonymous but your review may still be made public.

**Do you want your identity to be public for this peer review?**  For information about this choice, including consent withdrawal, please see our Privacy Policy.

Reviewer #1: No

Reviewer #2: No

**********

[NOTE: If reviewer comments were submitted as an attachment file, they will be attached to this email and accessible via the submission site. Please log into your account, locate the manuscript record, and check for the action link "View Attachments". If this link does not appear, there are no attachment files.]

To ensure your figures meet our technical requirements, please review our figure guidelines: https://journals.plos.org/plosone/s/figures

You may also use PLOS’s free figure tool, NAAS, to help you prepare publication quality figures: https://journals.plos.org/plosone/s/figures#loc-tools-for-figure-preparation.

NAAS will assess whether your figures meet our technical requirements by comparing each figure against our figure specifications.

# Author comment: Author response to Decision Letter 1

**DOI:** 10.1371/journal.pone.0353943.r002

25 Jun 2026

Please find specific responses to the review below (reviewer comments in italic):

-Michael C. Bazaco

1.Please ensure that your manuscript meets PLOS ONE's style requirements, including those for file naming. The PLOS ONE style templates can be found at

We have followed the style guide for the manuscript submission to the best of our understanding.

2. When completing the data availability statement of the submission form, you indicated that you will make your data available on acceptance. We strongly recommend all authors decide on a data sharing plan before acceptance, as the process can be lengthy and hold up publication timelines. Please note that, though access restrictions are acceptable now, your entire data will need to be made freely accessible if your manuscript is accepted for publication. This policy applies to all data except where public deposition would breach compliance with the protocol approved by your research ethics board. If you are unable to adhere to our open data policy, please kindly revise your statement to explain your reasoning and we will seek the editor's input on an exemption. Please be assured that, once you have provided your new statement, the assessment of your exemption will not hold up the peer review process.

Thank you for this clarification. When we chose this option, we were referring to the R code used for the model, so apologies for the confusion. All our data comes from the publicly available CDC Foodborne Disease Outbreak Surveillance System (FDOSS) and can be accessed at any time (https://www.cdc.gov/nors/about/fdoss.html). We have put a link to that data system into the manuscript (Line 76). In addition, we have made the R code for our modeling tool openly available as well. It is linked in the manuscript and that link is now live and open (Line 132).

3. It appears that your figure captions and supporting figure captions are the same. Please confirm if the files were intended as figures or supporting figures. If so, kindly remove the supporting table captions in your manuscript.

We were sure to upload the Figures as Figures, not Supporting Figures on resubmission and have removed the captions from the manuscript.

4. We are unable to open your Supporting Information file “S8 Trends in FBO R Code Package.zip”. Please kindly revise as necessary and re-upload.

We apologize for this. That attachment was the R package used to run the trend model described in the manuscript. In lieu of an attachment, we have made the R code public and embedded it into the revised manuscript draft (Line 132), attached.

5. If the reviewer comments include a recommendation to cite specific previously published works, please review and evaluate these publications to determine whether they are relevant and should be cited. There is no requirement to cite these works unless the editor has indicated otherwise.

This is not applicable for our resubmission.

6. Please review your reference list to ensure that it is complete and correct. If you have cited papers that have been retracted, please include the rationale for doing so in the manuscript text, or remove these references and replace them with relevant current references. Any changes to the reference list should be mentioned in the rebuttal letter that accompanies your revised manuscript. If you need to cite a retracted article, indicate the article’s retracted status in the References list and also include a citation and full reference for the retraction notice.

We will not be adding any additional references at resubmission.

Review Comments to the Author

Reviewer #1: Only comment is quality of images before downloading is hard to read. For publication it should be ensured that the higher quality images are those to be included in the text. Otherwise research is sound.

We are happy to work with the journal if there are any issues with the images during processing.

Reviewer #2: GENERAL: This is an interesting and well written manuscript detailing time trend analysis of outbreaks and associated illnesses for four different pathogens. Good use of negative binomial regression and intelligent presentation of results. I had very few comments on the manuscript, most of which related to understanding what data weren't included in the analysis and overall interpretation.

SPECIFIC:

Line 90: This description about the difference between the modelling approach taken in this paper and the IFSAC annual reports is unclear. Suggest re-wording this to make it crystal clear how they are different and what the implications are.

Thank you. We have added some additional language to better clarify the differences (lines 91-93)

Line 103: Regarding the definition of foodborne outbreaks, were these two or more illnesses required to be microbiologically confirmed?

These did not need to be laboratory confirmed. There did need to be confirmation of a common food source.

Line 107: What happened to multipathogen outbreaks, which increased over time due to institution of culture independent diagnostic testing (CIDT) at a similar time to whole genome sequencing? It would also be good to mention the impact of CIDT in the discussion in relation to increased detection of associated cases.

We note in the Data section of the methods that our analysis included only outbreaks where a single causal pathogen was identified. We have added some language to further emphasize that as well (Lines 80-81)

Line 107: How did you handle outbreaks implicating multiple foods or poorly defined foods, such as a meal containing multiple ingredients.

Outbreaks involving multiple foods were only included if a single contaminated ingredient was confirmed as the source of the outbreak or when all the ingredients in a multi-ingredient food could be classified into one of our categories (example fruit salad consisting only of various fruits). We have added some language to better explain that (Lines 82-85).

Line 156: I would like to see a summary of the included datasets for each pathogen at the beginning of the results section. I feel like it is important to say what was excluded too so we understand the total dataset under consideration.

All data used in this analysis came from the CDC Foodborne Disease Outbreak Surveillance System (FDOSS). We have added a note about this at the beginning of the Data section (Lines 76-78)

Line 205: Is this comparison between third and fourth time period actually for each one against the first (reference) time period, which is how you would normal consider results in negative binomial regression. I note that these types of comparisons are made elsewhere, so good to clarify.

This model was developed to allow for comparisons between any time period tested. This versatility is one of the strengths of the model, so comparisons are cited throughout, and indicated in the Figures.

Line 264: You mention here trends in foodborne illnesses, but these are really 'foodborne disease outbreaks and associated illnesses', as most foodborne illnesses aren't linked to outbreaks.

Thank you for this comment. We have adjusted the language surrounding the Minor and Parrett article, as well as the closing sentence of that paragraph (Lines 290-291 and Line 296)

Line 328: You note the potential impact of whole genome sequencing for increased salmonella outbreaks, which is interesting. I would encourage further exploration of this issue (maybe not in this manuscript), as despite its usefulness data on effectiveness of whole genome sequencing is very hard to come by.

Thank you for this comment. We agree that evaluating the continued impact of WGS (along with other tools such as CIDTs and metagenomics) in outbreak investigations and analysis is important and plan to continue to evaluate these impacts in future research.

Attachment Submitted filename: Bazaco et al - PLOS ONE Response to Reviewer Letter.docx

# Editor report: Decision Letter 1

**DOI:** 10.1371/journal.pone.0353943.r003

**Authors:** Csaba Varga

2 Jul 2026

Trends in foodborne outbreaks and outbreak-associated illnesses using a Bayesian trend model – United States, 1998–2018

PONE-D-26-18588R1

Dear Dr. Bazaco,

We’re pleased to inform you that your manuscript has been judged scientifically suitable for publication and will be formally accepted for publication once it meets all outstanding technical requirements.

Within one week, you’ll receive an e-mail detailing the required amendments. When these have been addressed, you’ll receive a formal acceptance letter and your manuscript will be scheduled for publication.

An invoice will be generated when your article is formally accepted. Please note, if your institution has a publishing partnership with PLOS and your article meets the relevant criteria, all or part of your publication costs will be covered. Please make sure your user information is up-to-date by logging into Editorial Manager at Editorial Manager® and clicking the ‘Update My Information' link at the top of the page. For questions related to billing, please contact billing support.

If your institution or institutions have a press office, please notify them about your upcoming paper to help maximize its impact. If they’ll be preparing press materials, please inform our press team as soon as possible -- no later than 48 hours after receiving the formal acceptance. Your manuscript will remain under strict press embargo until 2 pm Eastern Time on the date of publication. For more information, please contact onepress@plos.org.

Kind regards,

Csaba Varga, DVM MSc PhD

Academic Editor

PLOS One

Additional Editor Comments (optional):

Thank you for addressing all of the comments and suggestions.

Reviewers' comments:

# Editor report: Acceptance letter

**DOI:** 10.1371/journal.pone.0353943.r004

**Authors:** Csaba Varga

PONE-D-26-18588R1

PLOS One

Dear Dr. Bazaco,

I'm pleased to inform you that your manuscript has been deemed suitable for publication in PLOS One. Congratulations! Your manuscript is now being handed over to our production team.

At this stage, our production department will prepare your paper for publication. This includes ensuring the following:

* All references, tables, and figures are properly cited

* All relevant supporting information is included in the manuscript submission,

* There are no issues that prevent the paper from being properly typeset

You will receive further instructions from the production team, including instructions on how to review your proof when it is ready. Please keep in mind that we are working through a large volume of accepted articles, so please give us a few days to review your paper and let you know the next and final steps.

Lastly, if your institution or institutions have a press office, please let them know about your upcoming paper now to help maximize its impact. If they'll be preparing press materials, please inform our press team within the next 48 hours. Your manuscript will remain under strict press embargo until 2 pm Eastern Time on the date of publication. For more information, please contact onepress@plos.org.

You will receive an invoice from PLOS for your publication fee after your manuscript has reached the completed accept phase. If you receive an email requesting payment before acceptance or for any other service, this may be a phishing scheme. Learn how to identify phishing emails and protect your accounts at https://explore.plos.org/phishing.

If we can help with anything else, please email us at customercare@plos.org.

Thank you for submitting your work to PLOS ONE and supporting open access.

Kind regards,

PLOS ONE Editorial Office Staff

on behalf of

Dr. Csaba Varga

Academic Editor

PLOS One
